# Family medicine and physician reimbursement: a US perspective and the RCGP International and Overseas Network

**DOI:** 10.3399/bjgpopen18X101529

**Published:** 2018-04-07

**Authors:** Ayotunde Monica Uko

**Affiliations:** 1 Founder Member, RCGP International and Overseas Network, RCGP, London, UK; 2 Healthcare Consultant, La Crosse, WI, US

**Keywords:** Family Medicine, International and Overseas Network, USA, Royal College of General Practitioners

This article highlights a significant issue that will change the landscape of the US healthcare for decades to come: the Medicare Access and Children’s Health Insurance Program (CHIP) Reauthorization Act of April 2015 (MACRA).^1^ MACRA heralds a rare opportunity for the US healthcare delivery system to revamp the way it reimburses physicians, with a pivot from a volume-based payment system to one of quality and value. MACRA essentially is designed to reward or penalise physicians for patient care services that have been provided. This legislation established the Quality Payment Program (QPP) which is the overarching term for the two tracks through which Medicare payments would be made: merit-based incentive payment system (MIPS), and advanced alternative payment models (advanced APMs).^2^ The QPP aims to improve health outcomes, promote smarter spending, minimise burden of participation, and provide fairness and transparency in operations.^3^ It replaces Centers for Medicare & Medicaid Services legacy programmes for clinician quality reporting, including the physician quality reporting system (PQRS), the value-based payment modifier (VBPM), and the Medicare Electronic Health Records (EHR) Incentive Program for Eligible Professionals, to support the transition to value-based care and healthcare delivery system reform.^3–6^ Physicians and other providers are critical to the success of MACRA.^7^ Performance data collected in 2017 qualifies for payments in 2019.^7,8^ Box 1–2 outline the details of MIPS.

**Box 1.** MIPS performance categories

Performance year	Payment year	Positive/negative payment adjustment, %
2017	2019	±4
2018	2020	±5
2019	2021	±7
2020	2022	±9
2021	2023	±9

**Box 2.** MIPS adjustments sliding scale

Performance category	2017, %	2018, %	2019, %
Quality	60	50	30
Cost	0	10	30
Advancing care information	25	25	25
Improvement activities	15	15	15

MIPS adjusts payment to physicians based on performance in four performance categories: quality, based on the PQRS; cost, based on the VBPM; advancing care information, based on the Medicare EHR Incentive Program (meaningful use); and improvement activities, a new category.

While the MIPS model attempts to embrace value within the fee-for-service (FFS) reimbursement of providers, advanced APMs embrace a complete change to a 'bundle' or capitation-based reimbursement model.^9,10^ Advanced APMs are versatile and could be applied to a specific clinical condition, a care episode, or a population.^9^ Advanced APMs let practices earn more for taking on some risk related to their patients' outcomes. For instance, a physician could earn a 5% incentive payment and higher base payment rate updates than MIPS from 2026 onward for taking on more financial risk through an advanced APM.^9^

The advanced APM could be particularly vital for family practice in the US. In 2014, a survey showed that >880 million physician office visits were made by patients in the US.^11^Of these, 52.2% were primary care physician visits.^11^ Family doctors saw 21.8% of the total patient visits.^11^ It is evident from the data in this survey that family medicine plays a fundamental role within the healthcare system here in the US.^11^ In the current FFS system, family physicians have had to fight local turf battles over hospital privileges with internists, pediatricians, and obstetrician–gynecologists.^12^ In a recent American Academy of Family Physicians (AAFP) member survey, <20% of AAFP members have hospital privileges for routine obstetric delivery, and <60% have privileges for newborn care, down from 25.7% and 64.7%, respectively, in 1995.^12^ Advanced APM offers a bundle-based payment model that could reimburse family practitioners for the care of their patient populations more effectively compared to the traditional FFS model. Family physicians, through advanced APMs, would be allowed the flexibility of planning and coordinating the highest quality of individualised health care in a cost effective way. Research shows that half of the population spend very little on health care, while 5% of the population spends almost half of the total amount.^13^ Individuals within the top 5% spend about 17 times as much as those individuals within the bottom 50% of healthcare spenders.^13^ Higher spenders were identified among the older generation with a higher proportion of multiple expensive chronic conditions.^13^ For example, in one area of New Jersey, 1% of the population accounts for one-third of healthcare expenditure.^14^ The highest expense for insurers for one patient in that area was US$3.5 million.^14^ It is possible that the advanced APMs would offer a value-based model that enables family physicians to care for their population of patients with substantial primary care needs. This autonomy in care decisions could be a valuable avenue to attract new physicians into family medicine, as reimbursement could be evened out across the spectrum of practice.

Outside of MACRA, innovative models of financing that embrace capitation payments are already beginning to take hold in the healthcare space, and US family physicians can learn from them. An example can be seen in the model of care provided by Iora Health. Iora is a primary care organisation which offers valuable care to populations of patients with chronic conditions.^15^ A key aspect of Iora’s model is a form of 'capitation' payment, simplifying the process of reimbursement.^15^ Iora hires healthcare coaches and institutes home visits as a means to improve care coordination.^15^ The positive health outcomes in its patient population are a testament to its innovative model of care delivery.^15^

For all the potential for MACRA to make a difference in the current US healthcare system, it is imperfect. MIPS is designed to effectively identify winners and losers each year, irrespective of how much achievement has been made beyond stated thresholds.^7,8,16^ In addition to this, it is observed that there is a potential for incomes to plateau in future years.^7^ Over time, it is intended that, through MACRA, providers would make a shift from MIPS to the advanced APMs, which could offer greater income potential and higher financial risk for healthcare outcomes.^9,16^ There are also other contributory factors to US healthcare spending, and it will take much more than MACRA to make a real impact. For example, managing the complexity of an aging population and medical price inflation would require nuanced health policy formulation in addition to provider reimbursement reform.^17^

MACRA creates a crucial opportunity for the US health system to move from a volume-based reimbursement of physicians to a value-based one. A lot could be learnt from the UK experience with initiatives such as the Quality and Outcomes Framework, and the Royal College of General Practitioners International and Overseas Network could play a vital role in forming collaborations for this purpose.
